# Segmental resection of splenic flexure colon cancers provides an adequate lymph node harvest and is a safe operative approach – an analysis of the ACS-NSQIP database

**DOI:** 10.1007/s00464-021-08926-9

**Published:** 2022-01-01

**Authors:** Allison J. Pang, Daniel Marinescu, Nancy Morin, Carol-Ann Vasilevsky, Marylise Boutros

**Affiliations:** Division of Colon and Rectal Surgery, Jewish General Hospital, McGill University, Montréal, QC Canada

**Keywords:** Splenic flexure colon cancer, Segmental resection, Extended colectomy, Lymph node harvest, Morbidity, NSQIP

## Abstract

**Introduction:**

Fewer than 10% of colon cancers are found at the splenic flexure. A standard surgical approach to these cancers has not been defined. The goal of this study was to compare lymph node harvest and post-operative morbidity between segmental resection and formal left hemicolectomy for splenic flexure colon cancers.

**Method:**

Patients diagnosed with a splenic flexure cancer were identified from the 2012–2018 ACS-NSQIP colectomy-targeted database. Patients were categorized based on type of surgical resection – left hemicolectomy with colorectal anastomosis or segmental colectomy with colocolonic anastomosis. Demographic, clinicopathologic, and post-operative outcomes were compared between groups. Factors independently associated with lymph node harvest, operative time, and post-operative morbidity were investigated by linear and binomial logistic regression models.

**Results:**

A total of 3,049 patients underwent colectomy for a splenic flexure cancer. Of these, 83.6% had a segmental colectomy and 73% were performed by a minimally invasive approach. T- and N-stage did not differ between segmental and left hemicolectomy groups (*p* = 0.703 and *p* = 0.429, respectively). Inadequate nodal harvest (< 12 nodes) was infrequent and similar between the two procedures (7.4% vs. 9.1%, *p* = 0.13). Operative time was significantly shorter for segmental colectomy (213 ± 83.5 min vs. 193 ± 84.1 min, *p* < 0.0001) and major morbidity was similar between the two surgical techniques (8.4% vs. 8.9%, *p* = 0.75). After accounting for demographic, clinicopathologic, and operative factors, binomial logistic regression showed that type of procedure was not significantly associated with LN harvest (OR 0.80, 95%CI 0.54–1.17) or major morbidity (OR 1.17, 95%CI 0.36–3.81). However, on linear regression, segmental splenic flexure resection was associated with shorter operative time (estimate 20.29, 95%CI 12.61–27.97, *p* < 0.0001).

**Conclusion:**

Splenic flexure resection for colon cancer is associated with an adequate lymph node harvest. Compared to a formal left hemicolectomy, a segmental resection also has a shorter operative time with equivalent post-operative morbidity.

Splenic flexure colon cancers (SFCs) are relatively rare and account for less than 10% of all colorectal cancers [1, 2]. They are generally thought to have a poorer prognosis as they are often discovered at a more advanced stage and have an increased risk of bowel obstruction [1, 3]. Traditionally, SFCs are defined as tumors located within 10 cm of the splenic flexure edges or as arising from the colon between the distal third of the transverse colon and the proximal third of the descending colon [4].

There is currently no consensus on the optimal surgical approach to SFCs. As such, the debate revolves around three different procedures: segmental resection of the splenic flexure (segmental colectomy) with colocolonic anastomosis, subtotal colectomy (sometimes termed extended right hemicolectomy (ERH) in the literature) with ileocolonic anastomosis, or formal left hemicolectomy with colorectal anastomosis. Arguments in favor of one surgical procedure over another are largely based on the understanding of the vascularization and lymphatic drainage of the splenic flexure. However, the choice of operation is further complicated by the consideration of three important surgical outcomes: cancer-related survival, post-operative morbidity (specifically anastomotic leak), and functional results.

This has prompted some authors to evaluate the differences between these three surgical procedures and to also compare laparoscopic versus open approaches. The preference for segmental resection of the splenic flexure is relatively low in several centers [5], while others strongly advocate for this colon-sparing option as it avoids unnecessary resection of the middle colic vessels and preserves colonic length [6–10]. The current literature seems to suggest that a more limited colon resection is indeed safe and can still provide adequate oncologic outcomes; however, the few studies that do exist come from Europe and are based on single-center, retrospective studies. Currently, it would seem that subtotal colectomies/ERHs are largely unjustified in the elective setting as they are known to be associated with increased morbidity, slower recovery rates, and worse functional outcomes [9, 11, 12]. Therefore, the goal of this study was to use the ACS-NSQIP database to compare segmental colectomy (resection of the splenic flexure) and left hemicolectomy as surgical treatment options for SFCs. We hypothesized that resection of the splenic flexure for SFCs provides an oncologically adequate lymph node harvest while providing similar surgical outcomes compared to a formal left hemicolectomy.

## Methods and materials

The American College of Surgeons (ACS) National Surgical Quality Improvement Program (ACS-NSQIP) is a multi-institutional initiative that prospectively collects surgical outcomes data. It has been described previously [13]. Briefly, the ACS-NSQIP is a validated, risk-adjusted, outcomes-based program to measure and improve the quality of surgical care. Trained reviewers collect data, and it is this collective anonymous data that were used for the purposes of this project. A retrospective study was performed using this data set and an Institutional Review Board approval was obtained.

### Study population

Patients ≥ 18 years of age were identified from the 2012–2018 ACS-NSQIP colectomy-targeted patient user file (PUF). We included patients who had undergone an elective surgery for a malignant neoplasm of the splenic flexure for which nodal harvest results were available. These specific patients were identified using the following diagnostic codes from the International Classification of Diseases, Ninth and Tenth Revision (ICD-9 and ICD-10): 153.7, 153.2, 18.5, and 18.6. Patients with descending colon cancers were also included as these tumors are technically within the anatomical description of the splenic flexure. Transverse colon cancers, however, were not included as it was impossible to determine from the database if the cancer was within the distal third of the transverse colon. All emergency cases were excluded. The patients were then selected and categorized based on the type of surgical resection performed (either segmental colectomy or left hemicolectomy) using Current Procedural Terminology (CPT) codes. In the context of this study, a segmental splenic flexure resection was described as a partial colectomy with colocolonic anastomosis and the CPT codes used were “44140 and 44204”. A formal left hemicolectomy was described as a partial colectomy with colorectal anastomosis and the CPT codes used were “44145 and 44207”. Since CPT codes were used to identify patients, they included all forms of operative techniques. Patients who had concurrent procedures along with their colon cancer surgery were not excluded as it would be very unlikely that the concurrent procedure would be another colon resection in the context of a splenic flexure resection or left hemicolectomy. Patient information from the colectomy PUF was then linked to the general PUF to collect demographic, clinical, pathologic, and general operative and post-operative data. Pathologic evaluation was based on the 8^th^ edition UICC TNM classification. Only patients that had data in both the general and colectomy PUFs were included in the study.

### Outcomes

The primary outcome was adequacy of lymph node harvest (≥ 12 nodes) as a quality indicator for oncologic resection. This was another reason why subtotal colectomies/ERH were excluded from this study, as they would inherently be expected to have a higher number of lymph nodes in their specimen. The secondary outcomes studied were post-operative major morbidity and operative time. Major morbidity was defined by one or more of the following conditions: organ space or deep surgical site infections (SSIs), wound dehiscence, septic shock, sepsis, reintubation, reoperation, myocardial infarction, cardiac arrest, acute renal failure, pneumonia, deep vein thrombosis, and/or urinary tract infection (UTI).

### Statistical analysis

Categorical data were reported as frequencies and percentages and continuous data were reported as means and standard deviations. Normal distribution of continuous data was assessed. Univariate analysis of differences between surgical groups was performed by a two-tailed Student’s *t* test for continuous variables and a Pearson’s chi-square test for categorical variables. Binomial logistic or linear regressions were performed to determine factors associated with each outcome of interest. Covariates for the regression models were chosen a priori based on clinical subject knowledge. Statistical significance was set at *p* < 0.05. All statistical analyses were performed by R 3.4.1.

## Results

### Demographic and clinical characteristics of the study population

Between 2012 and 2018, a total of 3,049 patients were identified to have had an elective left hemicolectomy or segmental resection for a colon cancer of the splenic flexure. Of these patients, 499 (16.4%) underwent a left hemicolectomy and 2,550 (83.6%) patients underwent a segmental splenic flexure resection. Between these two surgical groups, there was a similar distribution of age, sex, body mass index (BMI), American Society of Anesthesiologists physical status classification (ASA classification), and medical comorbidities (Table 1). Approximately half of the patients from each intervention group had an ASA class of III or higher (55.9% v. 57.5%, *p* = 0.12). Compared to patients with a left hemicolectomy, more patients in the splenic flexure resection group were smokers (9.4% vs. 12.8%, *p* = 0.04), whereas more patients in the left hemicolectomy group had > 10% weight loss (5.0% vs. 3.0%, *p* = 0.04), although the event numbers for both of these variables were low.

**Table 1:** Demographic and clinical characteristics of the study cohort

n (%) or mean (SD)	Left hemicolectomy (partial colectomy with colorectal anastomosis)	Splenic flexure resection (segmental colectomy with colocolonic anastomosis)	*p*-value
Total population	499 (16.37)	2550 (83.63)	–
Age	64.5 ± 13.3	64.8 ± 13.0	0.648
Male sex	259 (51.9)	1397 (54.7)	0.257
Race			0.257
*White*	339 (67.9)	1675 (65.6)	
*Black or African American*	63 (12.6)	290 (11.3)	
*Other*	97 (19.4)	585 (22.9)	
ASA^*^			0.123
*I*	6 (1.2)	47 (1.8)	
*II*	197 (39.4)	1030 (40.3)	
*III*	3265 (53.1)	1342 (52.6)	
*IV*	29 (5.8)	125 (4.9)	
Smoking	47 (9.4)	328 (12.8)	0.038
BMI (kg/m^2^)^*^	29.5 ± 6.5	29.3 ± 6.8	0.527
Diabetes			0.999
*no insulin*	68 (13.6)	359 (14.0)	
*insulin*	30 (6.0)	159 (6.2)	
Hypertension	263 (52.7)	1417 (55.5)	0.259
CHF^*^	1 (0.2)	21 (0.8)	0.224
COPD^*^	28 (5.6)	121 (4.7)	0.479
Bleeding disorder	12 (2.4)	58 (2.2)	0.988
> 10% weight loss	25 (5.0)	79 (3.0)	0.043

*SD* = *standard deviation*

^*^ASA = *American Society of Anesthesiology; BMI* = *Body Mass Index; CHF* = *Congestive Heart Failure; COPD* = *Chronic Obstructive Pulmonary Disease*

### Tumor pathology and operative approach

Tumor pathology was based on TNM staging and was assessed for each patient (see Table 2). For the entire cohort, the frequency of Tis, T1, T2, T3, and T4 tumors was 0.5%, 11.5%, 13.6%, 52.8%, and 12.9%, respectively. With regards to lymph node involvement, the frequency of N0 and N ( +) disease for the entire cohort was 56.7% and 35.3%, respectively. The distribution of tumor pathology was the same between the two surgical groups (T-stage *p* = 0.70; N-stage *p* = 0.43). Despite a reasonably high number of locally advanced cancers, the proportion of open procedures was fairly low, with 28.6% of patients having undergone an open left hemicolectomy and 27.2% having undergone an open splenic flexure resection (*p* = 0.56). The event number for patients who were converted from laparoscopy to laparotomy was few (17.6%) and was also equally balanced between the two surgical groups (*p* = 0.62). The use of robotic technique was also low and similar between groups (11.2% vs. 8.0%, *p* = 0.12).

**Table 2:** Splenic flexure tumor pathology and operative approach

n (%) or mean (SD)	Left hemicolectomy (partial colectomy with colorectal anastomosis)	Splenic flexure resection (segmental colectomy with colocolonic anastomosis)	p-value
Total population	499 (16.37)	2550 (83.63)	–
Pathologic T-stage			0.703
*T0*	8 (1.6)	43 (1.6)	
*Tis*	1 (0.2)	14 (0.5)	
*T1*	64 (12.8)	288 (11.2)	
*T2*	74 (14.8)	340 (13.3)	
*T3*	260 (52.1)	1349 (52.9)	
*T4*	62 (12.4)	330 (12.9)	
Pathologic N-stage			0.429
*N0*	288 (57.7)	1440 (56.4)	
*N1*	111 (22.2)	626 (24.5)	
*N2*	51 (10.2)	289 (11.3)	
Operative approach (open vs. other)	143 (28.6)	695 (27.2)	0.563
Laparoscopic converted to open	46 (9.2)	215 (8.4)	0.625
Robotic	56 (11.2)	227 (8.9)	0.121

*SD* = *standard deviation*

### Oncologic outcomes

The primary outcome for this study was adequacy of lymph node harvest, as it is an important quality indicator for oncologic resection (Table 3). A resection with less than 12 resected lymph nodes is deemed oncologically inadequate. The proportion of patients with inadequate lymph node harvest was low and was not significantly different between left hemicolectomy and segmental resection (7.4% vs. 9.1%, *p* = 0.13). The mean total number of lymph nodes resected was higher for a left hemicolectomy (left hemicolectomy = 21.1 ± 12.2 nodes; segmental resection = 19.4 ± 9.8 nodes, *p* = 0.0078), but the actual mean lymph node harvest for both procedures was still well above the accepted 12 lymph node cut-off.

**Table 3:** Oncologic and Post-operative outcomes

n (%) or mean (SD)	Left hemicolectomy (partial colectomy with colorectal anastomosis)	Splenic flexure resection (segmental colectomy with colocolonic anastomosis)	p-value
Total population	499 (16.37)	2550 (83.63)	–
Nodal harvest (< 12 lymph nodes)	37 (7.4)	234 (9.1)	0.130
Lymph nodes harvested	21.1 ± 12.2	19.4 ± 9.8	0.0078
Anastomotic leak	20 (4.0)	96 (3.7)	0.486
Bleeding	34 (6.8)	182 (7.1)	0.871
Surgical site infection	19 (3.8)	109 (4.2)	0.723
Deep organ space infection	20 (4.0)	87 (3.4)	0.596
Septic shock	4 (0.8)	31 (1.2)	0.572
Myocardial infarction	3 (0.6)	15 (0.5)	0.999
Acute renal failure	2 (0.4)	6 (0.2)	0.855
Pneumonia	5 (1.0)	44 (1.7)	0.326
Deep vein thrombosis	3 (0.6)	25 (1.0)	0.724
Pulmonary embolism	4 (0.8)	20 (0.7)	0.999
Mortality	2 (0.4)	19 (0.7)	0.579
**Major morbidity	42 (8.4)	229 (8.9)	0.750
Operative time (mins.)	213.3 ± 83.5	192.9 ± 84.1	< 0.001
Length of stay (days)	5.3 ± 4.3	5.4 ± 4.5	0.805
Re-operation	24 (4.8)	96 (3.7)	0.331
Re-admission	39 (7.8)	214 (8.3)	0.339

*SD* = *standard deviation*

^**^Major morbidity = *organ space or deep SSIs, wound dehiscence, septic shock, sepsis, re-intubation, re-operation, myocardial infarction, cardiac arrest, acute renal failure, pneumonia, deep vein thrombosis, or urinary tract infection*

### Post-operative outcomes

The post-operative outcomes for each surgical group are outlined in Table 3. Only operative time was statistically different between groups – left hemicolectomy being the longer procedure (213 ± 84 min. vs. 193 ± 84 min., *p* < 0.001). Post-operative morbidity and mortality rates were equivalent between surgical procedures, including the NSQIP-defined major morbidity (8.4% vs. 8.9%, *p* = 0.75). Lastly, length of stay (5.3 ± 4.3 days vs. 5.4 ± 4.5 days, *p* = 0.80), reoperation (4.8% vs. 3.7%, *p* = 0.33), and re-admission (7.8% vs. 8.3%, *p* = 0.34) were similar between both intervention groups.

### Independent associations of surgical approach with lymph node harvest, post-operative morbidity, and operative time

After accounting for demographic, clinicopathologic, and operative factors, surgical approach was not independently associated with adequacy of lymph node harvest (OR 0.80, 95%CI 0.54–1.17) (Table 4). Similarly, surgical approach was also not independently associated with overall morbidity (OR 1.17, 95%CI 0.36–3.81) (data not shown). On the other hand, surgical approach was positively associated with operative time, segmental splenic flexure resection being the procedure with a significantly shorter operative time (estimate = 20.29, 95%CI 12.61–27.97, *p* < 0.0001) (data not shown).

**Table 4:** Multivariate analysis of demographic, clinical, pathologic, and operative variables according to primary outcome (lymph node harvest ≥ 12)

Variable	Odds ratio (OR)	95% confidence interval (CI)
Demographics		
^*^ Age	1.014	1.001–1.027
Male sex	1.085	0.824–1.430
ASA		
*I*	> 100	< 0.001- > 100
*II*	> 100	< 0.001- > 100
*III*	> 100	< 0.001- > 100
*IV*	> 100	< 0.001- > 100
Clinical		
* Smoking	1.595	1.096–2.321
BMI (kg/m^2^)	0.995	0.973–1.017
Diabetes		
*no insulin*	1.062	0.607–1.858
* insulin	1.811	1.294–2.536
* Hypertension	1.502	1.101–2.048
CHF	2.001	0.592–6.761
COPD	1.475	0.871–2.498
Bleeding disorder	1.344	0.619–2.917
> 10% weight loss	0.584	0.234–1.458
Pathologic		
Pathologic T-stage		
*T0*	0.527	0.043–6.523
*Tis*	1.247	0.081–10.168
*T1*	0.425	0.038–4.767
*T2*	0.262	0.023–2.921
*T3*	0.149	0.014–1.646
*T4*	0.121	0.011–1.364
Pathologic N-stage		
*N0*	1.438	0.137–15.144
*N1*	1.072	0.100–11.455
*N2*	1.414	0.1133–15.006
Nodal harvest	–	–
Operative		
Operative approach	0.796	0.542–1.169
Anastomotic leak	1.046	0.355–3.085
Bleeding	1.338	0.794–2.255
Pulmonary embolism	1.130	0.234–5.470
Mortality	1.728	0.392–7.627
Major morbidity	0.948	0.383–2.344
Operative time (mins.)	0.999	0.998–1.001
Length of stay (days)	1.015	0.979–1.052
Re-operation	0.839	0.319–2.206
Re-admission	1.000	0.572–1.748
Laparoscopy	0.795	0.557–1.135
Laparoscopic converted to open	0.648	0.364–1.156

^*^statistically significant

## Discussion

To the best of our knowledge this is the largest and first North American study which investigates the oncologic adequacy and safety of segmental colectomy for SFCs in the elective setting. Using the ACS-NSQIP database, patients treated surgically for SFC were identified. The adequacy of lymph node harvest and results of post-operative morbidity were compared between two different surgical interventions: left hemicolectomy with colorectal anastomosis versus splenic flexure segmental colectomy with colocolonic anastomosis. Subtotal colectomies/ERH were not included in this study for two reasons. One, we expected the lymph node harvest to be inherently higher due to the much longer length of colon being resected. Second, it is already well established that compared to less extensive colectomies, subtotal colectomies (or ERHs) have worse surgical outcomes with regards to post-operative recovery, overall morbidity, and colonic function [11]. As such, it would seem that this surgical procedure should generally be reserved for emergency surgeries related to obstructing SFCs.

Our study showed that compared to a formal left hemicolectomy, a segmental colectomy for SFC provides an oncologically adequate lymph node harvest and does not lead to any worse morbidity. Segmental colectomy offered the additional benefit of having a shorter operative time. Importantly, patients who underwent both surgical interventions had similar demographic and clinical characteristics, as well as a similar distribution of tumor pathology. We also found that there was a higher proportion of smokers in the segmental colectomy group. It is unlikely that smoking contributed to the selection of one technique over another because, of the two surgical procedures, it is the segmental colectomy group that would be the most susceptible to the negative effects of smoking (i.e., small vessel disease) as its anastomosis heavily relies on the marginal artery. Nevertheless, the lack of difference in anastomotic leak rates between interventions confirms that the increased number of smokers in the segmental colectomy group was not clinically significant.

There are two main limitations to this study. Firstly, since long-term oncologic data are not available in the NSQIP database, the present study was not designed to assess cancer-related survival. However, many other groups have shown that segmental colectomies for SFCs indeed result in similar cancer-related survival when compared to more extended colectomies [6, 9, 12, 14, 15]. One recent study looking at extended versus segmental colectomy for transverse colon cancers showed that extended colectomy was actually associated with poorer overall survival [16]. Secondly, there are inherent limitations to using a large database, specifically with regards to coding accuracy. For example, we were unable to differentiate between proximal and distal transverse colon cancers, and therefore, there was potential for missed proximal splenic flexure cancers.

Optimizing nodal harvest is also an important consideration in the surgical management of colon cancer. The lymphatic drainage of the splenic flexure remains fairly poorly understood, perhaps accounting for the ongoing debate regarding the optimal extent of colon resection for SFCs. Lymphatic mapping has helped surgeons with regard to this matter. Using lymphatic scintigraphy, Vasey et al. showed that the lymphatic drainage was strongly dominant along the LCA in 96% of patients compared to the left branch of the MCA (lt-MCA) [17], thus supporting the adequacy of segmental colectomy in most cases. Others have found that the lymph flow pattern of SFCs can also be centered around the root of the IMV [18]. In cases of suspected node negative disease, it was also found that there was rarely concurrent lymph flow along both the LCA and the lt-MCA [18].

Our results come at the heels of a very recently published retrospective Italian study that effectively showed that segmental resection is safe and provides similar overall and disease-free survival as other extended resections [19]. A nation-wide questionnaire was also recently presented to several prominent French surgical societies and cooperative groups to report on practices in France concerning the surgical management of SFCs [7]. Out of 190 surgeons, the preferred procedure was segmental colectomy (70%) with lymph node dissection limited to the LCA in the majority of cases. Only 29% of surgeons also performed a concurrent lymph node dissection around the middle colic vessels. After segmental colectomy, a left hemicolectomy was the next most preferred surgery (17%) followed by a subtotal colectomy (13%). Laparoscopy was employed in 63% of cases. Even though segmental resection was preferred, 29% of responders thought that SFCs had worse prognosis due to insufficient lymph node dissection; however, this opinion did not seem to influence the type of colectomy chosen to be performed.

The literature around SFCs and their optimal surgical intervention is limited and is based on a handful of case series, single-center retrospective studies, and matched case–control studies. While reviewing the literature, it is apparent that the definition of a left hemicolectomy is also not consistent among studies, forcing the reader to be vigilant of the extent of resection that is actually being described. There are even studies that define a left hemicolectomy as a mix of both segmental and extended colectomies, adding more confusion and bias to the study outcomes [20]. A single-center review of 98 patients who underwent either ERH or segmental colectomy showed similar post-operative morbidity and mortality [15], and also revealed that ERH was the most commonly practiced procedure at their center. Although there was no difference in overall and disease-free survival in this study, adequate nodal harvest was observed in only ~ 60% of segmental colectomies and in only 78% of ERHs. A subsequent study consisting of a large multi-center propensity-score matching (PSM) analysis compared all three operative approaches for SFCs – ERH, formal left hemicolectomy, and segmental colectomy [12]. The objective of retrieving at least 12 lymph nodes was achieved in about 85% of patients with no procedure-related differences. Although survival outcomes were all the same across the three groups, there was an observed increase in operative time, time to flatus, and hospital stay with progressive colonic resections. Also, ERH was specifically associated with significantly increased risk of post-operative ileus. An earlier PSM study by Arévalo et al.had also sought to compare the three procedures [21]. Although only a 2-by-2 comparison was performed, they did not show any major differences between surgical techniques. The percentage of patients with adequate lymph node harvest was not calculated in this study; however, there was surprisingly no difference in mean number of nodes resected. Lastly, another recent single-center Italian retrospective study demonstrated that segmental splenic flexure colectomy was the most popular surgical intervention for SFCs (55% of patients) practiced by their surgeons [6]. Of all three possible procedures, there was a similar number of patients with a lymph node harvest ≥ 12, in which each surgical group had a mean harvest of > 20 lymph nodes. Again, there was no difference in cancer-related survival or post-operative complications between surgical approaches.

In summary, all of these studies, including our own, demonstrate that segmental colectomy for SFCs results in an adequate lymph node harvest with a safe post-operative course. Moreover, despite inadequate lymph node harvests reported in older studies [15], the literature on segmental colectomies has never shown an inferior oncologic survival compared to extensive colonic resections. To further support the use of segmental colectomy, we are also cognizant that this procedure is associated with better functional results [11]. By preserving the right colon, the rectosigmoid junction, and the sympathetic nerve plexus around the IMA, there is a decreased potential for post-operative diarrhea, ureteric injuries, defecatory problems, and genitourinary issues.

## Conclusion

Based on the current literature, when compared to extended colectomy, a segmental colectomy of the splenic flexure is associated with better functional results without an increase in post-operative morbidity or a sacrifice in oncologic outcomes. Our study showed that segmental resection for SFCs is safe and provides an adequate oncologic lymph node harvest. Based on the ACS-NSQIP database, the majority of surgeons not only seem to prefer segmental colectomies of the splenic flexure, but also opt for a laparoscopic approach. Despite laparoscopic segmental resection of the splenic flexure being a potentially technically challenging surgery, operative time for segmental colectomies was still significantly shorter compared to formal left hemicolectomies. In conclusion, this large database study supports the elective resection of SFCs by segmental colectomy, proposing that procedures involving extended colon resections are not required.
